# Neuropathic pain in the community: More under-treated than refractory?

**DOI:** 10.1016/j.pain.2012.12.022

**Published:** 2013-05

**Authors:** Nicola Torrance, Janice A. Ferguson, Ebenezer Afolabi, Michael I. Bennett, Michael G. Serpell, Kate M. Dunn, Blair H. Smith

**Affiliations:** a Medical Research Institute, University of Dundee, Dundee, UK; b Division of Applied Health Sciences, University of Aberdeen, Aberdeen, UK; c Academic Unit of Palliative Care, University of Leeds, Leeds, UK; d School of Medicine, University of Glasgow, Glasgow, UK; e Institute of Primary Care & Health Sciences, University of Keele, Keele, UK

**Keywords:** Neuropathic pain, Chronic pain, Epidemiology, S-LANSS, Refractory

## Abstract

Best current estimates of neuropathic pain prevalence come from studies using screening tools detecting pain with probable neuropathic features; the proportion experiencing significant, long-term neuropathic pain, and the proportion not responding to standard treatment are unknown. These “refractory” cases are the most clinically important to detect, being the most severe, requiring specialist treatment. The aim of this study was to estimate the proportion of neuropathic pain in the population that is “refractory,” and to quantify associated clinical and demographic features. We posted self-administered questionnaires to 10,000 adult patients randomly selected from 10 general practitioner practices in 5 UK locations. The questionnaire contained chronic pain identification and severity questions, cause of pain, SF-12, EQ-5D, S-LANSS (Self-administered Leeds Assessment of Neuropathic Signs and Symptoms), PSEQ (Pain Self-Efficacy Questionnaire), use of neuropathic pain medications, and health care utilisation. These data were combined to determine the presence and characteristics of “refractory” neuropathic pain according to the defining features identified by a Delphi survey of international experts. Graded categories of chronic pain with and without neuropathic characteristics were generated, incorporating the refractory criteria. Completed questionnaires were returned by 4451 individuals (response rate 47%); 399 had “chronic pain with neuropathic characteristics” (S-LANSS positive, 8.9% of the study sample); 215 (53.9%) also reported a positive relevant history (“Possible neuropathic pain”); and 98 (4.5% of all Chronic Pain) also reported an “adequate” trial of at least one neuropathic pain drug (“Treated possible neuropathic pain”). The most refractory cases were associated with dramatically poorer physical and mental health, lower pain self-efficacy, higher pain intensity and pain-related disability, and greater health care service use.

## Summary

There is a significant proportion of chronic pain that is persistent and neuropathic, appears undertreated or untreated, and is associated with poor health and quality of life.

## 1 Introduction

Neuropathic pain was recently re-defined by the International Association for the Study of Pain (IASP) and graded as “possible,” “probable,” or “definite,” depending on the extent and results of neurological assessment [23,48]. Best current estimates of the population prevalence of neuropathic pain come from studies using validated screening tools that detect pain with possible neuropathic features [6,46]. Chronic pain with neuropathic features has been shown to affect around 7–8% of the general population. The proportion of these meeting the IASP classification of “possible” neuropathic pain, therefore requiring consideration of specific treatment or further assessment, is unknown. Similarly, the proportion experiencing significant, long-term neuropathic pain, not responding to standard treatment, is unknown, though this is perhaps the most clinically important and challenging subgroup.

The term “refractory neuropathic pain” has emerged in recent literature [34,43,45], and definitions and descriptions vary markedly. In a review of the epidemiology of refractory neuropathic pain, Taylor (2006) included specific neuropathic pain conditions and pain that was “persistent” [45]. The Scottish Medicines Consortium describes patients with refractory neuropathic pain, as those who “have not achieved adequate pain relief from, or have not tolerated, conventional first and second line treatments [35].” A more detailed definition was developed for use in a randomised controlled trial of a new drug regimen [43] and includes specific criteria related to duration of refractory neuropathic pain for at least 6 months, pain severity score of at least 40 mm on a 0–100 mm visual analogue scale, and nonresponse to usual care, including treatment with gabapentin, a tricyclic antidepressant, and a third potentially effective medication. Hansson et al. (2009) proposed a definition of “pharmacoresistant neuropathic pain” that would incorporate resistance to proven efficacious drugs, of appropriate duration, and with adequate dosage [20]. The authors concede that data are lacking to support an evidence-based approach to the definition of these terms and call for more studies and debate.

With refractory neuropathic pain defined and classified in a clinically and epidemiologically relevant way, it will be possible to identify those people in the community who experience this most severe neuropathic pain and who are in greatest need of specialist treatment. This will in turn allow an assessment of the scale of the problem, identification of risk factors for “refractoriness” (including those that are potentially modifiable), and the subsequent efficient targeting of management or prevention strategies. The apparent lack of such an agreed case definition for refractory neuropathic pain and a means of case ascertainment have hindered this endeavour. Recent research, however, involving an international Delphi survey of experts, defined “refractoriness” of neuropathic pain for epidemiological research [42], with the following key criteria: 1) there should have been a trial of treatment with at least 4 drugs of known effectiveness in neuropathic pain; 2) each of these drugs should have been tried for at least 3 months or until adverse effects prevent adequate dosage or continued treatment; 3) despite this treatment, the intensity of pain should not have been reduced by more than 30%, or should remain at a level of at least 5 on a 0–10 scale; and/or it should continue to contribute significantly to poor quality of life.

We have previously described the prevalence, distribution, and associated health of pain with neuropathic features in the general population, compared with those without chronic (neuropathic) pain [41,46]. The main aim of this study was to estimate the proportion of neuropathic pain in the population that is “refractory,” and to quantify associated clinical and demographic features. In a population-based study it is not practical to undertake detailed neurological assessment on a sufficient scale to identify “probable” or “definite” neuropathic pain, therefore we used the validated Self-administered Leeds Assessment of Neuropathic Signs and Symptoms (S-LANSS) questionnaire [4], supplemented by questions on diagnosis, to identify “possible” neuropathic pain. “Refractoriness” was determined according to the above criteria. We anticipated that the number of cases fully meeting all of these criteria, and therefore being ascertained as truly “refractory,” would be small. We therefore further aimed to measure the impact of each criterion (apparent resistance to treatment, pain intensity, pain-related quality of life) on overall prevalence and health outcomes. To do this, we developed a graded categorisation, with increasing “refractoriness” of neuropathic pain.

## 2 Methods

### 2.1 Sample selection

In the UK, around 96% of the population is registered with a general practitioner (family doctor, GP) [30]; a GP practice population therefore approximately equates a general population sample. This study surveyed a total of 10,000 individuals in 5 UK locations, with 2 GP practices in each locality, each generating a random sample of 1000 registered adult patients. In England, in Leeds, Lancaster, and Stafford, the National Institute for Health Research Clinical Research Network [29] provided support in identifying and recruiting practices and their patients. In Scotland, practices in Grampian and Glasgow participated, supported by the Scottish Primary Care Research Network [37]. Each practice’s electronic register was used to generate a random sample of patients aged over 18 years and the sample list was then screened by the GPs in each practice, to exclude patients in whom inquiry might be insensitive or inappropriate (for example, those with terminal illness or with severe learning difficulties). Each excluded patient was replaced by another, sampled randomly from the same practice register. The study was conducted between November 2010 and March 2011. One reminder letter and an additional copy of the questionnaire were posted to nonrespondents approximately 3 weeks after the first questionnaire.

The questionnaire was developed to categorise “refractory neuropathic pain” according to the expert definition generated by the Delphi study [42]. It included: 1) chronic pain identification questions and a measure of chronic pain with neuropathic characteristics (S-LANSS); 2) a relevant patient history; 3) questions on the number and duration of neuropathic pain medications tried; 4) pain intensity (using the Chronic Pain Grade [CPG]), and 5) health-related quality of life (SF-12 and EQ-5D). In order to further explore the impact of these levels of refractoriness, we also collected data on pain-related disability, pain self-efficacy, and health care utilisation.

### 2.2 Patient questionnaire

Questions were included on age, gender, smoking, marital and employment status, educational attainment, and home ownership (as a proxy for social class) [44]. These were similar to questions used in previous studies of chronic (neuropathic) pain in the community [14,46].

#### 2.2.1 Pain ascertainment/characteristics

Individuals with chronic pain were identified by affirmative answers to 2 questions: 1) Are you currently troubled by pain or discomfort, either all the time or on and off? 2) Have you had this pain or discomfort for more than 3 months? [21]. Identical case identification questions have been used in previous population-based research on chronic pain [13,14,46]. Participants who responded positively to both these questions were asked to indicate the site(s) of their chronic pain and the most troublesome site by selecting from a list that included: back; neck or shoulder; head, face or teeth; stomach or abdomen; arms or hands; chest; hips; and legs or feet [51]. Subsequent questions about pain, including the S-LANSS questions, related to the single most troublesome site of pain. The S-LANSS was used to identify pain with neuropathic characteristics (NC) [4]; a 7-item questionnaire, it includes 5 questions about pain characteristics and 2 self-examination items, with responses weighted to provide a maximum score of 24. A score of 12 or more has been found to have a positive predictive value for neuropathic pain of 76% (95% confidence interval [CI] 66.8–84.2%) when compared with classification by a pain specialist. Its validity and reliability in identifying pain with NC with this cut-off score in postal research has also been confirmed [4]. Individuals in this study who had an S-LANSS score ⩾12 were therefore identified as cases and described as having “Chronic pain with NC.”

Respondents were also asked to indicate whether any of the following common causes of pain had been diagnosed as the cause of the chronic pain they had identified in the screening questions: any type of neuralgia; back problems (such as slipped disc, back surgery or sciatica); diabetes; cancer or cancer treatment/chemotherapy; human immunodeficiency virus; muscle problems e.g. spasm, strains, tension; amputation of a limb; a surgical operation; arthritis; shingles; multiple sclerosis; stroke; an accident that damaged a nerve; or none of these. Responses to this question were dichotomised among those with S-LANSS score ⩾12 to “Not neuropathic pain” (i.e. muscle problems or arthritis) and “Possible neuropathic pain” (other possible neuropathic pain causes listed), and therefore, to indicate whether or not respondents had a “relevant history.”

The CPG, a 7-item instrument, was used to assess pain severity based on its intensity and pain-related disability [52]. The CPG questionnaire inquires about current, worst, and average pain in the previous 3 months and classifies its severity into 4 hierarchical grades: Grade I (low disability-low intensity), Grade II (low disability-high intensity), Grade III (high disability-moderately limiting), and Grade IV (high disability-severely limiting). The CPG is valid and reliable for use as a self-completion postal questionnaire in the UK general population [39]. Only those who responded positively to the case-screening questions, which identified whether chronic pain was present, were asked to complete the CPG questionnaire.

#### 2.2.2 Pain medication and health care use

Respondents were asked to indicate whether any neuropathic pain medications had been prescribed either currently or in the past. The list of pain medications was derived from recently published and rigorously evidence-based guidelines on the pharmacological management of neuropathic pain [1,16,31] and the British National Formulary [8]. These drugs were amitriptyline, carbamazepine, duloxetine, gabapentin, lidocaine patch, morphine, nortriptyline, oxcarbazepine, oxycodone, pregabalin, tramadol, and venlafaxine. Respondents were asked to indicate whether they were currently taking these medications and/or had taken them in the past, to specify how long they had taken each one, and the reasons they stopped taking them, for example, because of side effects or lack of effect. If they had taken the neuropathic pain drug for at least 3 months, this was considered an “adequate trial,” as was cessation because of side effects. In respondents who indicated that they had taken any neuropathic pain medication but for <3 months, or who selected “did not work” as the reason for cessation without an indication of the duration of treatment, this was not considered an “adequate” trial.

Respondents were also asked to indicate the number of consultations they had attended with a GP about their pain condition in the previous 6 months, if they had ever attended a pain management specialist/pain clinic, and to indicate which other health care professionals they had consulted for their chronic pain.

#### 2.2.3 Health-related quality of life

Assessment of health-related quality of life (HRQoL) in all respondents was based on the Medical Outcomes Short-Form 12 scale (SF-12), a validated self-administered tool for measuring health status derived from the SF-36 [53]. The SF-12 has been used in large general population questionnaire studies of chronic pain [2,9,26] and in specific neuropathic pain conditions, such as zoster-related pain [5]. SF-12 scores can be calculated in 8 health domains: physical functioning, role physical, bodily pain, general health, vitality, social functioning, role emotional, and mental health. The scores are summarised into 2 component scores – mental health (MCS) and physical health (PCS), with scores ranging from 0 (worst possible health state) to 100 (best possible health state). The participants’ scores on the SF-12-MCS and SF-12-PCS were further categorised into tertiles: lowest third = “poor” HRQoL, middle third = “moderate” HRQoL, and highest third = “good” HRQoL. A similar approach has been used by Nicholl et al. (2009) [33].

The EQ-5D is a generic measure of health status and defines health in terms of 5 dimensions: mobility, self-care, usual activities (work, study, housework, family, or leisure), pain or discomfort, and anxiety or depression, and is well validated in population studies [15,24]. A preference-based set of weights (or algorithm) is used to calculate a single EQ-5D index-based utility score of HRQoL, anchored by 1 (full health) and 0 (equal to death), with some states being worse than death (<0) [22]. In addition, the EQ-5D also includes a numerical rating scale ranging from 0 (worst imaginable health state) to 100 (best imaginable health state), on which respondents can rate their current health.

#### 2.2.4 Pain self-efficacy questionnaire

The Pain Self-Efficacy Questionnaire (PSEQ) measures pain cognition and self-confidence in performing functional and social activities, despite the presence of pain [32]. It has high intra-rater reliability, internal consistency, and stability on retest. The PSEQ includes 10 items, each with a 7-point scale, where 0 equals “not at all confident” and 6 equals “completely confident,” and a total score is calculated by summing the scores for each of the 10 items, yielding a maximum possible score of 60, with higher scores reflecting stronger self-efficacy beliefs. There is good evidence that higher self-efficacy about managing pain is associated with more positive treatment outcomes, higher return to work rates, better adherence, more effective control of pain and effect, and better prognosis [28].

### 2.3 Data analysis

Data were analysed using PASW Statistics for Windows (version 18.0; SPSS Inc, Chicago, IL, USA). Simple descriptive statistics and cross-tabulations were used to estimate the prevalence of Chronic pain with NC (i.e., S-LANSS score of 12 or more), “Possible neuropathic pain” (S-LANSS score ⩾12 and a relevant diagnosis from the above list), and “Refractory” neuropathic pain (“possible” cases fulfilling the above criteria for refractoriness) [42]. Given the stringency of the criteria for refractoriness, we also explored degrees of “refractoriness,” including those with an adequate trial of 0, 1, and 2 or more neuropathic pain medications, and different combinations of pain severity and HRQoL. In addition, to explore the relative effects of pain severity and pain characteristics, we explored sub-groups of those with “severe” pain (average pain intensity ⩾7/10) in those with and without NC.

For the purposes of analysis and comparison, graded categories of chronic pain were identified (Table 1): “Chronic pain,” “Chronic pain without NC,” “Chronic pain with NC,” “Possible neuropathic pain,” “Untreated possible neuropathic pain,” “Treated possible neuropathic pain,” and “Refractory possible neuropathic pain.” “Chronic pain with and without NC” are mutually exclusive groups, as are “Untreated possible neuropathic pain” and “Treated neuropathic pain.” For all other chronic pain groups, individuals could be in more than one due to the reported pain characteristics deemed important for identifying neuropathic pain and “Refractory possible neuropathic pain” by international experts [42].

Chi-squared tests were used to test for associations and statistical significance between categorical sociodemographic variables, and *t*-tests were used to explore the mean difference in normally distributed continuous variables. All reported *P* values were from 2-sided tests, and a *P* value < 0.05 was used to denote statistical significance.

### 2.4 Ethical approval

The study was approved by North of Scotland Research Ethics Committee, REC reference number 09/S0802/103.

## 3 Results

A total of 10,000 questionnaires were mailed by the Primary Care Research Networks on the authors’ behalf. Of these, 347 were returned as undelivered or unable to be completed due to illness or learning disability. A total of 4541 completed questionnaires were returned, giving an overall corrected response rate of 47%. Response rates varied between practices and ranged from 35% to 58%. The response rate also varied with age and gender, with nonresponders younger than responders (mean [SD] age, 44.5 [17.4] years vs 53.4 [16.9] years, *P* < 0.001) and women more likely to respond than men across all practices (57.5% vs 42.6%; *P* < 0.001). These patterns were similar in each practice (data available on request).

**Fig. 1:**
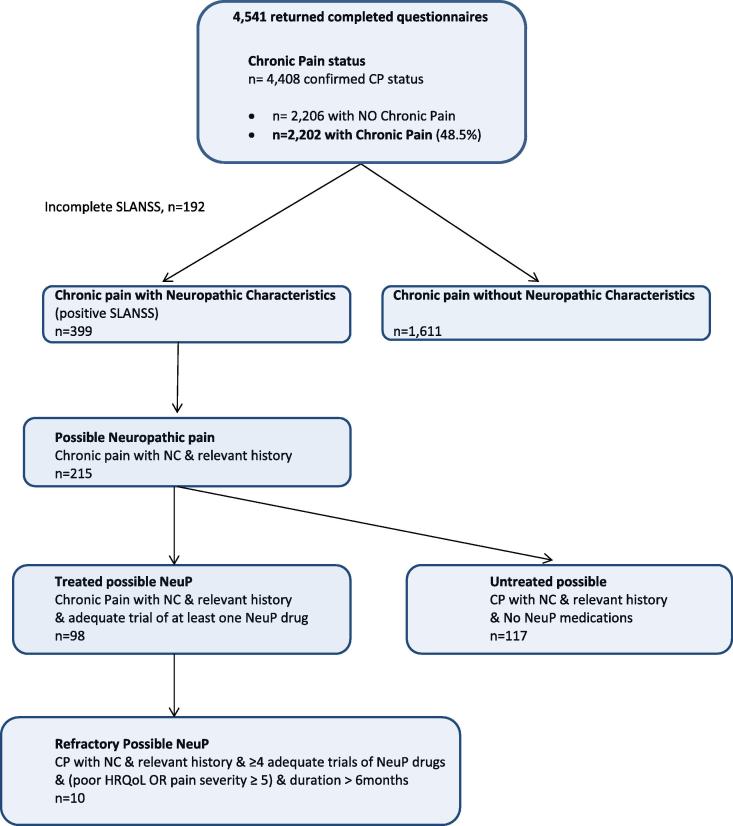
Outline of respondents chronic pain status/groups for analysis. S-LANSS, Self-administered Leeds Assessment of Neuropathic Signs and Symptoms; NeuP, NeuP, neuropathic pain; NC, neuropathic characteristics; HRQoL, health-related quality of life.

Of the 4541 returned questionnaires, 4498 individuals completed both of the screening questions for chronic pain status (43 individuals did not complete the 2 case-identification questions for chronic pain and were excluded from further analysis). Chronic pain was reported by 2202 (48.5%; 95% CI 47.0–49.9%). S-LANSS questionnaires were incomplete in 192 of these individuals. Therefore, the study sample for analysis included 4306 individuals with complete data: 2296 respondents with no chronic pain and 2010 respondents with any chronic pain; which comprised 1611 individuals with Chronic pain without NC, and 399 with Chronic pain with NC (S-LANSS positive). The 399 respondents who reported Chronic pain with NC represented 8.9% (399/4451) of the study sample, and 18.1% (399/2202) of those reporting any chronic pain Fig. 1.

### 3.1 Characteristics and severity of pain with neuropathic characteristics and associated quality of life and pain self-efficacy

There were significant differences in all of the measured sociodemographic characteristics between respondents reporting Chronic pain with NC and those reporting Chronic pain without NC (Table 2) except for age (mean [SD] 56.0 [15.4] years vs 56.3 [15.3] years, *P* = 0.673). Individuals with Chronic pain with NC were more likely to be women, no longer married, and living in council-rented accommodation than individuals whose chronic pain did not have NC. They were also more likely to be unable to work due to illness or disability, to have no educational qualifications, and to be smokers. However, amongst those individuals who reported Severe Chronic pain (pain intensity ⩾7/10), the differences in gender and education were not significant (Table 2).

Table 3 shows the impact of chronic pain with and without NC, and also those reporting severe pain. Significantly more of those with Chronic pain with NC reported Grades III and IV of the CPG, indicating greater disability due to pain (50.7% vs 21.9%, *P* < 0.001), and among those with Severe chronic pain, those with NC were more likely to be disabled as a result of their pain (68.2% vs 47.0%, *P* < 0.001) than those with Chronic pain without NC. Although Chronic pain with NC was associated with greater pain duration compared to Chronic pain without NC, the duration of Severe chronic pain was similar in both groups. Chronic pain with NC was associated with significantly lower SF-12 scores in all domains, and with significantly lower utility index scores for EQ-5D and poorer pain self-efficacy than Chronic pain without NC.

### 3.2 Characteristics of “possible” and “refractory” neuropathic pain including relevant history, duration, impact, and treatment of neuropathic pain

The graded categories of chronic pain with neuropathic characteristics, incorporating the features of “refractoriness,” are shown in Table 4 and Fig. 1. Of the 399 reporting Chronic pain with NC, 215 (53.9%) also reported a relevant medical history of a possible medical condition known to feature neuropathic pain (Table 4). In this group of those with “Possible neuropathic pain” (n = 215), neuralgia was the most commonly reported cause (n = 69, 32.1%), followed by back problems such as slipped disc, back surgery, or sciatica (n = 67, 31.1%), a surgical operation (n = 45, 20.9%), an accident that damaged a nerve (n = 35, 16.3%), diabetes (n = 21, 9.8%), cancer or cancer treatment/chemotherapy (n = 9, 4.2%), shingles (n = 5, 2.3%), stroke (n = 5, 2.3%), multiple sclerosis (n = 4, 1.9%), and amputation of a limb (n = 3, 1.4%); 50 (12.5%) of these respondents indicated more than one cause of possible neuropathic pain. Of these 215 people with “Possible neuropathic pain,” 117 also indicated that they had never had an “adequate trial” of neuropathic pain medications (“Untreated possible neuropathic pain”); the other 98 had had an adequate trial of one or more of these drugs (“Treated possible neuropathic pain”) (Table 4). Of individuals who had had an adequate trial of at least 2 neuropathic pain drugs (n = 52), all but 2 respondents also reported an average pain intensity of at least 5/10, and 34 (8.5% of S-LANSS positive) also had “poor” HRQoL according to their PCS and MCS scores in the SF-12. There were 10 true “Refractory” cases (positive S-LANSS, relevant history, adequate trial of 4 or more neuropathic pain medications, pain intensity at least 5/10, or poor HRQoL).

Comparisons of selected subgroups of those with “Possible” and “Possible refractory” neuropathic pain representing those who had and had not been treated with adequate trials of neuropathic pain medications are shown in Table 5, representing grades of “refractoriness.” We were unable to conduct statistical testing between these groups, as they are not mutually independent (i.e., individuals belong to more than one group, see Table 1). Pairwise comparisons between “Untreated possible neuropathic pain,” “Treated possible neuropathic pain,” and “Refractory possible neuropathic pain” are shown in Table 6.

The mean age of those in the “Possible,” “Untreated Possible,” and “Treated Possible” neuropathic groups ranged from 55.3 to 57.6 years, with the “Refractory Possible” neuropathic group slightly younger, with a mean age of 51.7 years (SD 11.5) (Table 5). The proportions of male and female responders are similar in the graded groups. In responses to both of the HRQoL questionnaire instruments, there was a decline in scores with each gradation of “refractoriness,” that is, addition of characteristics from the agreed expert definition [42]. These differences were more apparent in responses to the SF-12 PCS and in the proportion with severe pain-related disability measured by the CPG. The “Refractory Possible” neuropathic pain group was found to have the poorest physical and mental health component scores in the SF-12, the lowest EQ-5D index scores, lowest pain self-efficacy, and the highest pain severity.

Comparing those who had been treated (i.e., prescribed an adequate trial of at least one neuropathic pain drug) with those who reported no neuropathic pain treatment, we found significant differences in SF-12 PCS and MCS scores, EQ-5D, and PSEQ scores, with those with “Untreated possible neuropathic pain” reporting higher HRQoL and self-efficacy scores, indicating better overall health (Table 6). The much poorer scores in all these measures among those with “Refractory possible” neuropathic pain were found to be significant (with the exception of the SF-12 MCS) despite the small numbers in this group.

### 3.3 Use of health services

The highest proportion of people with chronic pain who had *not* visited their GP in the previous 6 months regarding the illness or medical condition that caused their pain were those with Chronic pain without NC (50%; n = 790) (Table 7). Almost 80% (n = 75) of those with “Treated possible” neuropathic pain had attended more than once. Half of those with “Refractory possible” neuropathic pain (n = 5) had attended their GP more than 4 times in the previous 6 months.

**Fig. 2:**
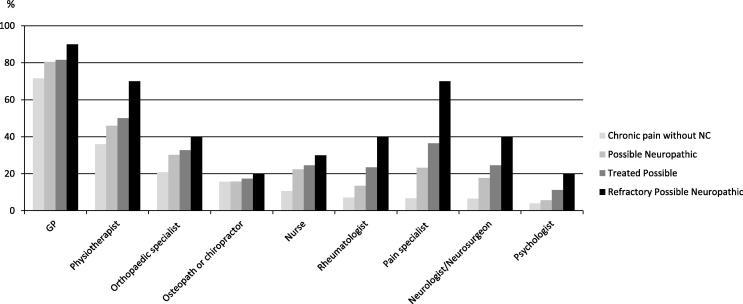
Proportion of pain respondents who are currently or have ever consulted a health care professional for their chronic pain (n = 1569 individuals without neuropathic characteristics [NC]; n = 215 Possible Neuropathic^a^; n = 98 Treated Possible^b^; n = 10 Refractory Possible neuropathic^c^). ^a^S-LANSS (Self-administered Leeds Assessment of Neuropathic Signs and Symptoms) positive and relevant history and no neuropathic pain (NeuP) medications. ^b^S-LANSS positive and relevant history and adequate trial of at least one NeuP medication. ^c^Possible NeuP (S-LANSS positive and relevant history) and adequate trial of 4 or more drugs and poor QoL *OR* pain severity >5 and pain duration more than 6 months.

Referral to a pain specialist was most common in the “Refractory possible” neuropathic pain group (70%, n = 7) compared to 6.7% (n = 106) of those reporting Chronic pain without NC. Among those with Severe chronic pain (⩾7/10), individuals who reported pain with NCs were significantly more likely to have attended a pain management specialist (or been to a pain clinic) than those reporting Severe Chronic pain *without* NC (21.8% [n = 46] vs 10.5% [n = 50], *P* < 0.001). Of health care professionals consulted for pain, the GP was the most commonly reported by all groups, followed by a physiotherapist (Fig. 2).

## 4 Discussion

This study uniquely incorporates the essential features of an international consensus on measuring the epidemiology of refractory neuropathic pain [42] in a questionnaire survey of a large general population sample. “Possible” neuropathic pain is relatively common, accounting for about 10% (215/2202) of people with chronic pain and 53.9% of those reporting neuropathic characteristics on the S-LANSS. It is associated with poorer physical, psychological, and social health than Chronic pain without NC, though all chronic pain is associated with poor health [38]. Although truly “refractory” neuropathic pain, as defined by international experts, is relatively uncommon (affecting just 5% of those with possible neuropathic pain), it is associated with severe pain and pain-related disability and extremely poor health in all dimensions; this is despite numerous attempts at pharmacological treatment and high use of health services. While a relatively low response rate prevents accurate estimates of prevalence, it is apparent that there is a significant proportion of people in the community with persistent neuropathic-type pain that remains untreated or undertreated, with no adequate trials of effective medication. Until this proportion of patients has been exposed to adequate treatment (defined within the Introduction), we are unable to estimate the proportion of patients with truly refractory neuropathic pain.

This study comprises a large dataset derived from a random sample of adults generated from GP practices located across the UK. The questionnaire contained well-validated questionnaire instruments, including the S-LANSS, which enabled us to categorise respondents as having chronic pain with and without neuropathic characteristics [4]. The relatively low response rate is similar to those in previous surveys of pain prevalence [7,27], and an increasingly common problem in epidemiological research [17,25,27]. The questionnaire was sent to a number of areas of high deprivation (particularly in Glasgow), and low socioeconomic status is known to be associated with poorer response rates [17,50]. The main concern with low response is that it introduces the potential for responder bias, making it difficult to generalise prevalence estimates to the population. In this paper we therefore focused on the proportion of all chronic pain represented by possible (refractory) neuropathic pain, and on comparisons between well-defined pain subgroups. Despite the response rate, we found that the proportion of the sample reporting any chronic pain was very similar to that found in a previous study using an identical case definition and with a response rate of 81% [14]. We also found that chronic pain with NC was reported by a similar proportion (8.9%) and similar sociodemographic associations as in previous research with higher response rates [6,46].

We are likely to have overestimated “Possible neuropathic pain,” as some of the diagnoses included in the list might include nonneuropathic pain (e.g., back pain). However, these reported chronic pains may have a neuropathic contribution, and can be considered as a spectrum, “more or less neuropathic” [3]. It is likely, given the positive S-LANSS, that those individuals in this group had an important neuropathic contribution to their pain; without clinical examination it is impossible to comprehensively identify “possible,” “probable,” or ”definite” neuropathic pain, and a population-based questionnaire survey can only approximate [48]. Although we relied on self-report for the categorisation of respondents, the instruments we used were all well validated [4,14,15,52,53]. The questions on the self-reported diagnoses of causes of pain, and the drug history were not formally validated. We made these as user-friendly as possible, with a comprehensive checklist, and tested them in a pilot study. Previous research based on electronic prescribing records has described in detail the changes in prescribing for neuropathic pain [19]. With our current data, we cannot confirm the validity of responses to pain diagnoses or drug history, and further research, based on reviewing medical records, is necessary to examine this.

We have been conservative in our approach to identifying those individuals who had an “adequate trial” of a neuropathic pain medication. It was apparent that, on some occasions, these drugs were not prescribed for chronic possible neuropathic pain, for example, opioids, tramadol, and morphine, where respondents indicated that they had taken these drugs for an acute episode of pain, for example, postsurgical. These were appropriately excluded from criteria for an adequate trial. Conversely, a number of individuals indicated that they had taken neuropathic pain medication “in the past,” but did not specify the length of time these were prescribed/taken for; again, we excluded these people from the “adequate trial” criteria. It is possible this may have resulted in misclassification. We did not ask about dosage of the neuropathic pain medications, as this was felt to be too onerous for respondents, possibly further diminishing the response rate, and unlikely to be accurately reported. Future research, with more detailed drug history, is required to explore this. Levels of prescribing in studies in general practice settings is generally low in patients with presumed neuropathic pain [2,18,47] (identified by screening questionnaires, such as the S-LANSS and DN4) and in patients with specific neuropathic pain diagnoses, such as postherpetic neuralgia (PHN) [12]. In our study, three quarters of respondents with “Possible neuropathic pain” (S-LANSS positive and relevant history) had consulted their GP regarding their pain in the previous 6 months, suggesting that opportunities to improve management and treatment may have been missed. This is likely to be for a combination of reasons, including education and resources. Recent guidelines describe the full range of drug treatments available [1,16] and, in the UK, evidence-based guidelines focus on primary care/nonspecialist settings [31]. This work highlights the scale of under- or untreated neuropathic pain, and the opportunity to improve its management in primary care, where the great majority of these pain patients exist.

Previous research has found the presence of neuropathic pain to be associated with poor health in every dimension: physical, psychological, and social [11,41]. In this study, among individuals reporting Chronic pain with NC, we found similar physical and mental component summary scores (PCS/MCS) for the SF-12 questionnaire to those in a French nationwide general population survey [2]. Our EQ-5D utilities were generally lower (indicating poorer quality of life) than those in some other chronic conditions, including cancer, heart failure, chronic obstructive pulmonary disease, type 2 diabetes, Parkinson disease, and stroke [11], although they are comparable to other studies of neuropathic pain conditions: Chronic pain with NC (mean EQ-5D utility index 0.47) was comparable to a Canadian study of patients with peripheral neuropathy determined by the Toronto Clinical Neuropathy Score [36], and also to mean pooled utility scores for diabetic neuropathy (0.61), PHN (0.61), and mixed neuropathic pain (0.43) [11]. Furthermore, in our study, the “Refractory possible” neuropathic pain group had the lowest mean EQ-5D health utility index score (mean 0.01), even lower than those found in studies of failed back surgery syndrome (0.15) and central neuropathic pain (0.23) [11]. An EQ-5D index score of 0 is “equal to death” [22], highlighting the extreme detrimental and debilitating burden that refractory neuropathic pain exerts on these individuals, encompassing all aspects of HRQoL. This is confirmed by the very low self-efficacy scores, indicating that patients in this group do not feel confident to perform their normal activities in the presence of this pain. Other research has found the health impact of any neuropathic pain appears to be worse than that of nonneuropathic pain of equivalent intensity [40,41], and the burden is more dependent on its intensity than the cause [11]. Attal et al. (2011) suggest that it is the particular features, the strange and unpleasant signs and symptoms of this type of pain, and the distressing and unpleasant nature of the symptoms themselves that impact on quality of life [2].

In this cross-sectional study, it is impossible to determine the temporal nature of associations, particularly those linking refractory pain with quality of life and pain self-efficacy. Our findings indicate that it is possible that medical treatment makes these worse; however, the greater intensity and disability scores suggest that people with worse neuropathic pain are more likely to seek and receive adequate treatment (Tables 5 and 6), and that this will explain the link. Further research is required. We did not include questions about the use of drugs that were not specific to neuropathic pain in the list used to determine refractoriness. While this is consistent with current evidence [1,16] and the Delphi survey [42], it may have excluded those with mild neuropathic pain, perhaps controlled by paracetamol (acetaminophen) or other simple analgesics. These individuals are likely to have been included in the “Untreated Possible neuropathic pain” group. Note, though, that those with untreated neuropathic pain still had relatively poor HRQoL and severe pain, and many are likely to benefit from further assessment with a view to effective treatment.

Health care resources were widely used by all those with chronic pain. Overall, the GP was most commonly the health professional who was currently consulted about pain by all respondents, and similar results were reported across Europe by Breivik et al. [7]. There was greater use of health services in both primary care and specialist health care in the most refractory groups, where 70% had attended a pain management specialist, and had been prescribed a number of neuropathic pain medications (n = at least 4 drugs in the “Refractory possible” neuropathic pain group). We have identified a number of patients who report highly disabling pain and for whom adequate pain management does not appear to have been achieved. There is limited research on the effectiveness of standard psychological or multidisciplinary treatment programmes specifically designed for patients with chronic neuropathic pain, although it has been suggested that it is reasonable to extrapolate from successful trials in other types of chronic pain [10,49].

### 4.1 Conclusions

This study has found that truly refractory neuropathic pain (as defined by an international group of experts) is relatively uncommon. However, we have shown that there are many more individuals whose neuropathic pain has been unsuccessfully treated, and who have increasingly severe pain-related disability, poor quality of life, and high health care use. Most importantly, there is a significant proportion of chronic pain in the community that is apparently persistent and neuropathic, but that appears undertreated or untreated.

## Conflict of interest statement

This study was supported by an unrestricted educational grant from Pfizer UK Ltd. NT, JF, EA and KD have no conflicts of interest. BS has received occasional lecture and consultancy fees, on behalf of his Institution, from companies involved in the manufacture of drugs used in treating neuropathic pain. MS has received research support, consulting fees, or honoraria in the past 3 years from Astellas, Astra Zenica, Grünenthal, GW Pharmaceuticals, Lilly, NAPP and Pfizer. MB has received consultancy fees and lecturer honoraria from Pfizer, Astellas and Grunenthal in the last 3 years The authors assert no personal pecuniary or other conflict of interest in the writing of this article. No writing assistance was utilized in the production of this manuscript.

## Figures and Tables

**Table 1:** Graded categories of chronic pain for analysis.

‘*Chronic pain*’. Affirmative responses to the two chronic pain screening questions, (i) are you currently troubled by pain or discomfort, either all the time or on and off? (ii) Have you had this pain or discomfort for more than three months? [20] • *‘Chronic pain without neuropathic characteristics’*. Chronic pain (as above) and S-LANSS score of <12 • *‘Chronic pain with neuropathic characteristics’*. Chronic pain and S-LANSS positive (S-LANSS questionnaire score ⩾12) • *‘Possible neuropathic pain’*. Chronic pain; S-LANSS positive; and reported history of possible cause of neuropathic pain • *‘Untreated possible neuropathic pain’*. Chronic pain, S-LANSS positive; reported history of possible cause of neuropathic pain; and no reported history of neuropathic pain medications • *‘Treated possible neuropathic pain’*. Chronic pain, S-LANSS positive; reported history of possible cause of neuropathic pain; and reported adequate trial of at least one neuropathic pain medication • *‘Refractory possible neuropathic pain’*. Chronic pain; S-LANSS positive; and reported history of possible cause of neuropathic pain; and reported adequate trial of four or more drugs; and poor QoL *OR* pain severity greater than 5/10; and pain duration more than six months [41]

S-LANSS, Self-administered Leeds Assessment of Neuropathic Signs and Symptoms.

**Table 2:** Sociodemographic characteristics of respondents with No chronic pain and (Severe) Chronic pain with and without neuropathic characteristics (NC), n (%).

	No chronic pain (n = 2296)	Chronic pain with NC (n = 399)	Chronic pain without NC (n = 1611)	*P* value^a^	Severe chronic pain with NC^b^ (n = 218)	Severe chronic pain without NC^b^ (n = 487)	*P* value^a^
*Age, n (%)*							
18–39 years	654 (28.5)	60 (15.3)	234 (14.7)	0.326	30 (14.0)	67 (13.9)	0.112
40–59 years	928 (40.4)	167 (42.5)	622 (39.0)		97 (45.3)	180 (37.4)	
60+ years	692 (30.1)	166 (42.2)	740 (46.4)		87 (40.7)	234 (48.7)	

*Gender*							
Men	1016 (44.3)	146 (36.8)	684 (42.5)	0.043	68 (31.3)	186 (38.3)	0.092
Women	1280 (55.7)	251 (63.2)	925 (57.5)		149 (67.7)	300 (61.7)	

*Marital status*							
Never married	382 (16.6)	55 (14.0)	166 (10.4)	<0.001	31 (14.4)	50 (10.3)	0.005
Living as married	1614 (70.3)	245 (62.2)	1194 (74.5)		127 (59.1)	348 (71.6)	
No longer married	290 (12.6)	94 (23.9)	243 (15.2)		57 (26.5)	88 (18.1)	

*Housing tenure*							
Owned/mortgaged	1914 (85.4)	261 (66.4)	1321 (82.6)	<0.001	128 (59.8)	365 (75.4)	<0.001
Council rent	190 (8.5)	92 (23.4)	188 (11.8)		66 (30.8)	96 (19.8)	
Private rent/other	178 (7.8)	40 (10.2)	91 (5.7)		20 (9.3)	23 (4.8)	

*Employment*							
Employed	1483 (64.6)	151 (38.4)	774 (48.3)	<0.001	67 (31.3)	191 (39.6)	<0.001
Retired	562 (24.5)	137 (34.9)	638 (39.9)		75 (35.0)	204 (42.3)	
Unable to work	28 (1.2)	77 (19.6)	70 (4.4)		57 (26.6)	45 (9.3)	
Not employed/other	212 (9.2)	28 (7.1)	119 (7.4)		15 (7.0)	42 (8.7)	

*Education*							
No qualifications	323 (14.1)	115 (29.6)	345 (21.7)	<0.001	72 (34.1)	159 (33.1)	0.179
Secondary school/equivalent	933 (40.6)	165 (42.5)	599 (37.7)		92 (43.6)	183 (38.1)	
Higher education	1010 (44.0)	108 (27.8)	644 (40.6)		47 (22.3)	138 (28.7)	

*Smoking*							
Smoker	372 (16.2)	105 (26.4)	264 (16.4)	<0.001	66 (30.4)	107 (22.0)	0.003
Ex-smoker	632 (27.5)	109 (27.5)	580 (36.1)		49 (22.6)	168 (34.6)	
Never smoked	1288 (56.1)	183 (46.1)	761 (47.3)		102 (47.0)	211 (43.4)	

a Chi-squared test; comparisons between “Chronic pain with NC” vs “Chronic pain without NC” and “Severe chronic pain with NC” vs “Severe chronic pain without NC.”

b Severe pain = average pain intensity score ⩾7/10.

**Table 3:** Clinical and associated features associated with No Chronic pain and (Severe) chronic pain, with and without neuropathic characteristics (NC).

	No chronic pain (n = 2296)	Chronic pain with NC (n = 399)	Chronic pain without NC (n = 1611)	*P* value	Severe chronic pain with NC (n = 218)^a^	Severe chronic pain without NC (n = 487)^a^	*P* value
*SF-12, mean (SD)*							
Physical function	53.0 (8.0)	37.9 (13.0)	46.1 (11.6)	<0.001^b^	34.4 (12.1)	41.0 (13.0)	<0.001
Role physical	53.7 (7.2)	38.3 (12.3)	46.0 (11.2)	<0.001	34.5 (11.4)	40.2 (12.4)	<0.001
Bodily pain	54.3 (7.0)	33.3 (12.2)	43.0 (11.3)	<0.001	29.0 (10.7)	35.9 (12.1)	<0.001
General health	52.1 (8.8)	36.7 (13.4)	44.3 (11.7)	<0.001	33.6 (13.2)	39.5 (12.4)	<0.001
Social function	52.2 (8.5)	38.5 (13.5)	47.0 (11.6)	<0.001	34.9 (13.1)	41.8 (13.1)	<0.001
Role emotional	51.6 (8.6)	39.4 (14.8)	47.2 (11.9)	<0.001	35.8 (15.3)	42.0 (14.4)	<0.001
Vitality	53.7 (8.8)	43.5 (11.2)	47.8 (10.2)	<0.001	41.9 (11.1)	44.0 (10.9)	<0.001
Mental health	51.9 (8.9)	42.7 (11.7)	48.2 (10.5)	<0.001	40.3 (12.0)	44.6 (11.5)	<0.001
Physical component score	54.0 (7.0)	35.7 (12.8)	44.5 (11.8)	<0.001	32.0 (11.6)	38.6 (12.8)	<0.001
Mental component score	51.9 (7.0)	43.7 (12.8)	48.8 (11.2)	<0.001	41.1 (13.0)	45.2 (12.7)	<0.001
EQ-5D index score, mean (SD)	0.93 (0.13)	0.47 (0.34)	0.70 (0.25)	<0.001	0.33 (0.35)	0.55 (0.32)	<0.001
EQ-VAS, mean (SD)	85.3 (13.5)	59.7 (24.0)	73.4 (19.3)	<0.001	51.8 (24.6)	64.3 (22.4)	<0.001

*Chronic pain grade, n (%)*							
Grade I	–	63 (16.6)	681 (44.3)	<0.001^c^	3 (1.4)	11 (2.3)	<0.001
Grade II	–	128 (33.8)	518 (33.7)		63 (30.3)	239 (50.6)	
Grade III	–	76 (21.1)	189 (12.3)		56 (26.9)	111 (23.5)	
Grade IV	–	112 (29.6)	148 (9.6)		86 (41.3)	111 (23.5)	

*Pain duration (mo), n (%)*							
<6	–	16 (4.0)	107 (6.7)	0.005^c^	7 (3.3)	28 (5.8)	0.290
6–12	–	40 (10.1)	194 (21.1)		19 (8.8)	49 (10.2)	
12–36	–	84 (21.2)	417 (26.1)		40 (18.6)	102 (21.3)	
⩾36	–	256 (64.6)	880 (55.1)		149 (69.3)	301 (62.7)	

*Pain intensity, n (%)*							
Mild (1–3)	–	29 (7.4)	405 (25.8)	<0.001^c^	–	–	
Moderate (4–6)	–	145 (37.0)	677 (43.1)		–	–	
Severe (7–10)	–	218 (55.6)	487 (31.0)		–	–	
Pain self-efficacy, mean (SD)	–	32.99 (17.3)	44.81 (15.0)	<0.001^c^	26.4 (16.0)	34.7 (16.3)	<0.001

a Severe pain = average pain intensity score ⩾7/10.

b Chi-squared test; comparisons between “Chronic pain with NC” vs “Chronic pain without NC” and “Severe chronic pain with NC” vs “Severe chronic pain without NC.”

c *t*-Test comparisons between “Chronic pain with NC” vs “Chronic pain without NC” and “Severe chronic pain with NC” vs “Severe chronic pain without NC.”

**Table 4:** Graded categories of chronic pain with neuropathic characteristics, incorporating features of “refractoriness” [41].

		n	% of all chronic pain (n = 2202)	% of S-LANSS positive (n = 399)
B	S-LANSS positive	399	18.1	–
C	S-LANSS positive and relevant history^a^	215	9.8	53.88
D	S-LANSS positive and relevant history and NO neuropathic pain medications	117	5.3	29.32
E	S-LANSS + relevant history + an adequate trial^‡^ of one or more neuropathic pain medications	98	4.45	24.56
	S-LANSS + relevant history + adequate trial ⩾2 NeuP drugs	52	2.36	13.0
	S-LANSS + relevant history + adequate trial of ⩾2 NeuP drugs + pain severity ⩾5^b^	50	2.27	12.5
	S-LANSS + relevant history + ⩾2 adequate trials of NeuP drugs + poor quality of life^c^	34	1.5	8.5
F	S-LANSS + relevant history + ⩾4 adequate trials of NeuP drugs + (poor quality of life c or pain severity ⩾5) + duration >6 months	10	0.45	2.0

S-LANSS, Self-administered Leeds Assessment of Neuropathic Signs and Symptoms; NeuP, neuropathic pain.

a Relevant history – reported cause of pain dichotomised in those with S-LANSS score ⩾12 to “Not neuropathic pain” (i.e., muscle problems or arthritis) and “Possible neuropathic pain” (other possible neuropathic pain causes listed).

b Adequate trial – each of these drugs should have been tried for at least 3 months or until adverse effects prevent continued treatment.

c Poor Quality of Life = within the lowest tertile of scores for SF-12 PCS (Physical Component Score) and MCS (Mental Component Score).

**Table 5:** Clinical and associated features of graded possible refractory neuropathic pain.

	A	B	C	D	E	F
	Chronic pain without NC (n = 1611)	Chronic pain with NC (n = 399)	Possible NeuP^a^ (n = 215)	Untreated possible NeuP^b^ (n = 117)	Treated possible NeuP^c^ (n = 98)	Refractory possible NeuP^d^ (n = 10)
Age, mean (SD)	56.3 (15.3)	56 (15.4)	56.6 (14.8)	57.6 (16.3)	55.3 (12.7)	51.7 (11.5)

*Proportion of respondents by gender, n (%)*						
Female (n = 2456)	925 (37.7)	251 (10.2)	136 (5.5)	66 (2.7)	70 (2.9)	8 (0.3)
Male (n = 1846)	686 (37.2)	148 (8.0)	79 (4.3)	51 (2.8)	28 (1.5)	2 (0.1)

*SF-12, mean (SD)*						
PCS	44.5 (11.8)	35.7 (12.8)	33.9 (12.7)	36.8 (12.4)	30.3 (12.2)	18.1 (4.4)
MCS	48.8 (11.2)	43.4 (12.8)	43.8 (11.7)	46.6 (11)	40.5 (11.7)	40.3 (9.5)
EQ-5D index score	0.70 (0.25)	0.47 (0.34)	0.45 (0.3)	0.53 (0.3)	0.35 (0.3)	0.01 (0.06)
PSEQ, mean (SD)	44.8 (15)	33 (17.3)	32.2 (17.1)	37.8 (16.4)	25.3 (15.5)	12.6 (7.9)
Average pain intensity	5.2 (2.4)	6.7 (2.2)	6.7 (2.1)	6.2 (2.1)	7.3 (2)	8.3 (1.4)
Pain-related disability (CPG III & IV)	337 (21.9)	188 (49.6)	109 (53.2)	44 (40.0)	65 (68.4)	10 (100)

NeuP, neuropathic pain; PCS, Physical Component Score; MCS, Mental Component Score; PSEQ, Pain Self-Efficacy Questionnaire; CPG, Chronic Pain Grade.

a Self-administered Leeds Assessment of Neuropathic Signs and Symptoms (S-LANSS) positive and relevant history.

b S-LANSS positive and relevant history and no NeuP medications.

c S-LANSS positive and relevant history and adequate trial of at least one NeuP medication.

d Possible NeuP (S-LANSS positive and relevant history) and adequate trial of 4 or more drugs and poor QoL *OR* pain severity >5 and pain duration more than 6 months.

**Table 6:** Comparisons of health-related quality of life, general health, and pain self-efficacy between “Untreated,” “Treated,” and “Refractory” possible neuropathic pain (for pain groups see Table 1), Mean (SD).

	D	E	Mean difference	95% CI	*P*-value
	Untreated possible NeuP^2^ (n = 117)	Treated possible NeuP^3^ (n = 98)			
SF-12 PCS	36.8 (12.4)	30.3 (12.2)	6.5	3.1–9.9	<0.001
SF-12 MCS	46.6 (11.0)	40.5 (11.7)	6.1	3.0–9.3	<0.001
EQ-5D	0.53 (0.3)	0.35 (0.3)	0.1	0.1–0.3	<0.001
PSEQ	37.8 (16.4)	25.3 (15.5)	12.5	8.1–17.0	<0.001

	D	F			
	Untreated possible NeuP^2^ (n = 117)	Refractory possible^4^ (n = 10)			
SF-12 PCS	36.8 (12.4)	18.1 (4.4)	18.7	10.9–26.6	<0.001
SF-12 MCS	46.6 (11.0)	40.3 (9.5)	6.2	−0.8–13.3	0.084
EQ-5D	0.53 (0.3)	0.01 (0.06)	0.5	0.3–0.7	<0.001
PSEQ	37.8 (16.4)	12.6 (7.9)	25.3	14.4–36.3	<0.001

NeuP, neuropathic pain; CI, confidence interval; PCS, Physical Component Score; MCS, Mental Component Score; PSEQ, Pain Self-Efficacy Questionnaire.

**Table 7:** Consultations with the GP for pain, n (%).^a^

	Chronic pain without NC (n = 1569)	Possible neuropathic^b^ (n = 215)	Treated possible^c^ (n = 98)	Refractory possible neuropathic^d^ (n = 10)
*Consultations about pain condition with their GP in past 6 months*				
None	790 (50.4)	51 (25.1)	19 (20.2)	2 (20)
1–3	623 (39.7)	95 (46.8)	38 (40.4)	3 (30)
4–6	101 (6.3)	34 (16.7)	19 (20.2)	2 (20)
More than 6	55 (3.4)	23 (11.3)	18 (19.1)	3 (30)

GP, general practitioner; NC, neuropathic characteristics.

a Values are valid n (%).

b Self-administered Leeds Assessment of Neuropathic Signs and Symptoms (S-LANSS) positive and relevant history and no neuropathic pain (NeuP) medications.

c S-LANSS positive and relevant history and adequate trial of at least one NeuP medication.

d Possible NeuP (S-LANSS positive and relevant history) and adequate trial of 4 or more drugs and poor QoL *OR* pain severity >5 and pain duration more than 6 months.
